# Dual Antiplatelet Therapy After Elective Coronary Artery Bypass Graft and Its Impact on Clinical Outcomes

**DOI:** 10.1016/j.atssr.2024.11.012

**Published:** 2024-12-11

**Authors:** Iftikhar Ali Ch, Faisal Latif, Ahmad Usmani, Jeffrey Garrett, Pei-Tzu Wu, Mashal Tahirkheli, Rahat Jamal, Steven Miller, Naeem Tahirkheli

**Affiliations:** 1 South Oklahoma Heart Research, Oklahoma City, Oklahoma; 2 SSM Health Saint Anthony Hospital, Oklahoma City, Oklahoma; 3 University of Oklahoma, Oklahoma City, Oklahoma; 4 Southern California University of Health Sciences, Whittier, California

## Abstract

**Background:**

Dual antiplatelet therapy (DAPT) has demonstrated survival benefits in patients undergoing coronary artery bypass grafting (CABG) for acute coronary syndrome. The impact of DAPT in patients undergoing elective CABG remains underexplored.

**Methods:**

This single-center retrospective observational study reviewed all CABG procedures performed between 2012 and 2015. The primary outcome measured was the difference in mortality (all-cause and cardiovascular) between patients receiving aspirin monotherapy and those on DAPT. Secondary outcomes included post-CABG acute coronary syndrome, cerebrovascular accident, and major adverse cardiovascular events.

**Results:**

Among the 1828 patients who underwent elective CABG surgery, those who received DAPT had lower rates of all-cause mortality (odds ratio [OR], 0.43; 95% CI, 0.28-0.66; *P* < .001) and cardiovascular mortality (OR, 0.30; 95% CI, 0.16-0.56; *P* < .001) than aspirin monotherapy recipients. The overall survival rates were 93% and 97% for the aspirin monotherapy and DAPT groups, respectively (*P* < .001). The groups showed similar incidences of post-CABG acute coronary syndrome (OR, 1.14; 95% CI, 0.71-1.85; *P* = .629), post-CABG cerebrovascular accident (OR, 0.80; 95% CI, 0.45-1.43; *P* = .457), and major adverse cardiovascular events (OR, 0.73; 95% CI, 0.52-1.01; *P* = .061). Although DAPT patients experienced a higher rate of in-hospital major bleeding than aspirin monotherapy patients (OR, 1.48, 95% CI, 1.22-1.79; *P* < .001), the transfusion requirement was similar between the 2 groups (OR, 1.06, 95% CI, 0.77-1.46; *P* = .710).

**Conclusions:**

The use of DAPT in elective CABG patients was associated with significantly higher survival rates and reduced incidences of all-cause and cardiovascular mortality than aspirin monotherapy.

## Visual Abstract

**Figure:**
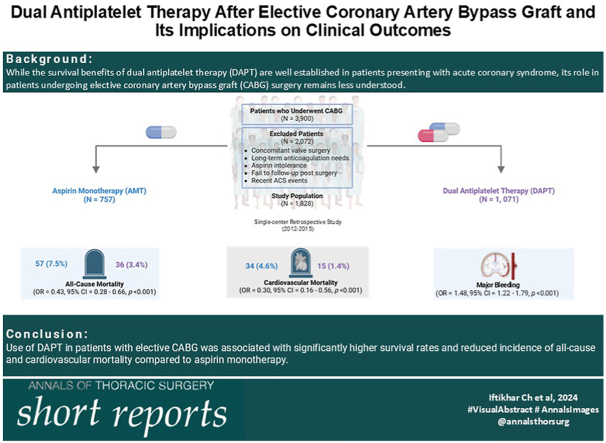

**In Short**

▪ This study found that dual antiplatelet therapy after elective coronary artery bypass grafting was associated with significantly lower all-cause and cardiovascular mortality compared with aspirin.
▪ These findings suggest that dual antiplatelet therapy may offer survival benefits in elective coronary artery bypass grafting patients, similar to those who undergo surgery after acute coronary syndrome.

Antiplatelet therapy plays a crucial role in managing patients undergoing coronary artery bypass graft (CABG) surgery.^1^ Although the benefits of dual antiplatelet therapy (DAPT) are well documented for individuals who present with acute coronary syndrome (ACS), evidence regarding its efficacy in patients who have undergone elective CABG surgery remains limited.^2^^,^^3^ The 2016 American College of Cardiology/American Heart Association Guidelines and the 2017 European Society of Cardiology Focused Update on DAPT use in coronary artery disease both recommend 12 months of DAPT after CABG for ACS patients.^4^^,^^5^

The role of DAPT following in patients with elective CABG remains uncertain. There is low-level evidence supporting DAPT use in such patients to enhance saphenous vein graft patency^4^; however, dedicated randomized clinical trials adequately powered for survival benefits in this population are lacking. Notably, although patients who undergo bypass surgery after coronary stenting are typically advised to resume DAPT postoperatively to maintain stent patency,^4^^,^^5^ the evidence supporting DAPT use after elective CABG without recent stent placement is sparse.^3^ A recent single-center study from Korea encompassing 5782 patients revealed that DAPT use was associated with a lower adjusted risk of cardiovascular death or myocardial infarction at 5 years among ACS patients, but not among those who presented with stable angina.^6^

Given the current scarcity of data on DAPT in patients undergoing CABG electively, we conducted a retrospective analysis of clinical outcomes in this group of patients.

## Patients and Methods

### Study Design

This retrospective observational study assessed the efficacy of DAPT in patients undergoing CABG surgery electively (no history of ACS within previous 12 months) at Oklahoma Heart Hospital, one of the largest cardiac surgery centers in the United States. The Institutional Review Board approved the study. The antiplatelet therapy received at discharge was used to categorize patients into 2 groups: DAPT and aspirin monotherapy (AMT). DAPT was defined as a combination of aspirin and a P2Y12 receptor antagonist for at least 1 month after CABG. Patients who underwent concomitant valve surgery, had long-term anticoagulation needs, aspirin intolerance, a history of an ACS event within 1 year before CABG, or did not follow-up at the institute after surgery were excluded (Figure, A).

**Figure:**
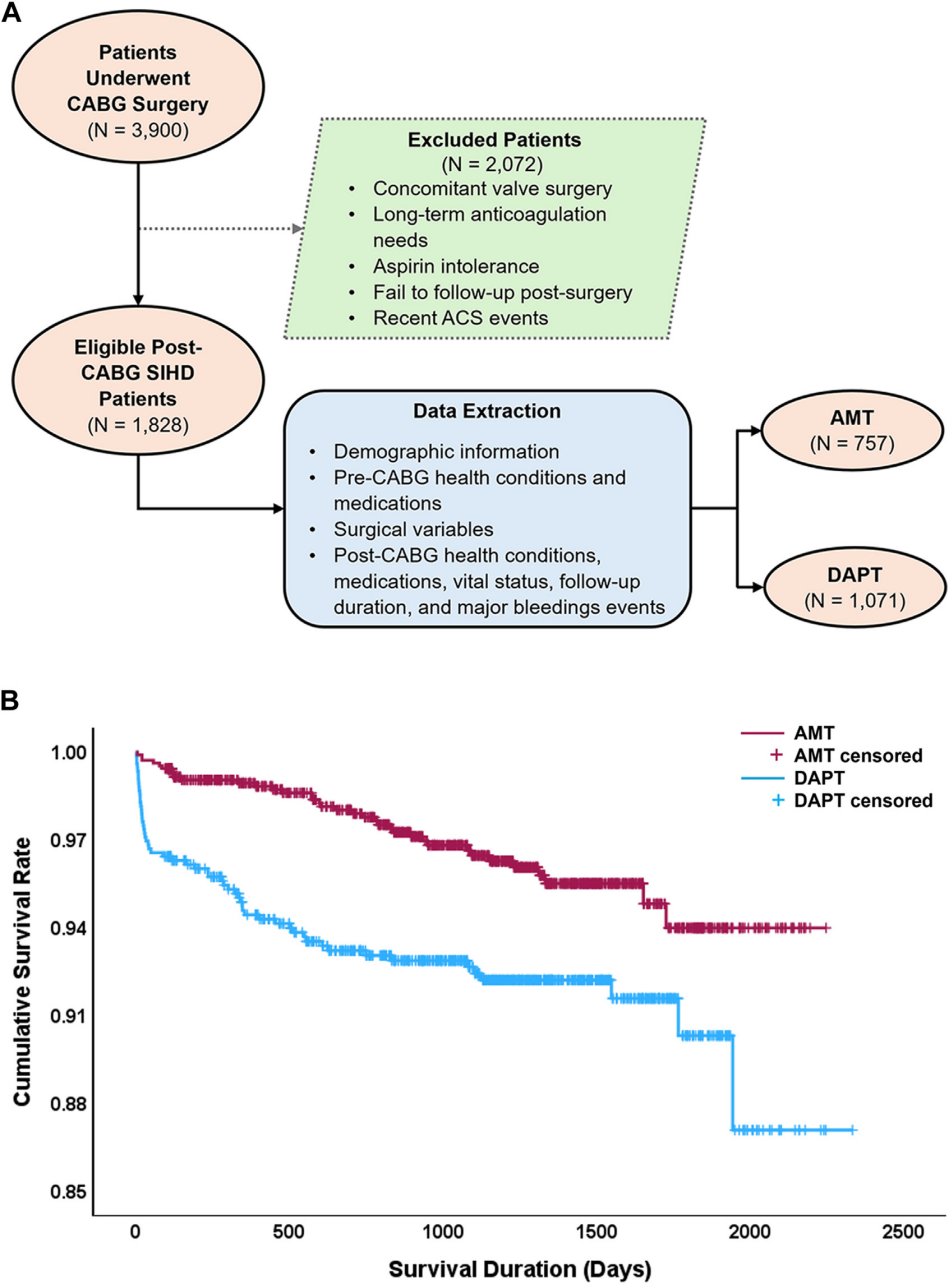
(A) Flowchart of the study design. (AMT, aspirin monotherapy; CABG, coronary artery bypass grafting; DAPT, dual antiplatelet therapy; SIHD, stable ischemic heart disease.) (B) Kaplan-Meier curves of the AMT and DAPT groups. The estimated mean survival time (post-CABG all-cause mortality-free time) was 2150.3 ± 25.5 days (95% CI, 2100.4-2200.2 days) in the AMT group and 2170.8 ± 13.4 days (95% CI, 2145.3-2196.2 days) in the DAPT group. The mean survival time was significantly longer in the DAPT group (log-rank test, χ^2^_(1)_ = 14.79, *P* < .001) (ACS, acute coronary syndrome).

### Outcome Measures

Cardiovascular mortality included deaths from various cardiac causes, whereas major adverse cardiac events were defined as a composite of ACS, cerebrovascular accident (CVA), and cardiovascular mortality. Post-CABG ACS was identified using appropriate International Classification of Diseases codes and was confirmed by electrocardiogram findings and abnormal laboratory values. CVA was defined as focal neurologic deficits lasting at least 24 hours or leading to death. Major bleeding was defined as a drop in hemoglobin >5 g/dL, hemorrhagic cardiac tamponade, intracranial hemorrhage, or any bleeding causing hemodynamic instability.

### Statistical Analysis

Descriptive statistics and frequencies were calculated for continuous and categorical variables. Continuous variables were compared between groups using the Mann-Whitney *U* test. Categorical variables were compared between groups using the χ^2^ test. Survival rates were compared between groups using Kaplan-Meier analysis. The Cox proportional hazards regression model was estimated using the forward conditional method based on the likelihood ratio. The incidence proportions and hazard ratios of various outcomes were compared between the AMT and DAPT groups. Statistical significance was set at a 2-tailed *P* value <.05. All analyses were conducted with SPSS 29.0 software (IBM Corp, Armonk, NY).

## Results

The study included 1828 patients who underwent elective CABG surgery between 2012 and 2015 after the specified exclusion criteria were applied. Most patients were prescribed DAPT (n = 1071 [58.6%]), with the remainder receiving AMT (n = 757 [41.4%]) (Figure, A). Baseline characteristics of both treatment groups are detailed in Table 1. Patients who died within 48 hours of surgery (n = 11) were excluded from further analysis.

**Table 1:** Patient Demographics, Comorbidities, and Medications

Variables	AMT	DAPT	*P* Value
	(n = 757)	(n = 1071)	
Age, y	65.9 ± 0.4	65.0 ± 0.3	**.043**
Male sex	582 (76.9)	785 (73.3)	.09
Race/ethnicity			.484
Caucasian	670 (88.5)	925 (86.4)	
African American	25 (3.3)	43 (4.0)	
Native American	26 (3.4)	47 (4.4)	
Hispanic	21 (2.8)	29 (2.7)	
Asian	7 (0.9)	13 (1.2)	
Pacific Islander	0	1 (0.1)	
Multiple	1 (0.1)	7 (0.7)	
Unknown	7 (0.9)	6 (0.6)	
Body mass index, kg/m^2^	30.75 ± 0.21	30.71 ± 0.18	.952
Smoker	377 (49.8)	593 (55.5)	**.017**
Comorbidities			
Hypertension	666 (88.0)	939 (87.7)	.885
Stroke	54 (7.1)	101 (9.4)	.088
Chronic obstructive pulmonary disease	190 (25.1)	287 (26.8)	.418
Hyperlipidemia	536 (70.8)	756 (70.6)	.958
Diabetes mellitus	311 (41.1)	427 (39.9)	.628
Chronic kidney disease	130 (17.2)	142 (13.3)	**.023**
Myocardial infarction	123 (16.2)	198 (18.5)	.236
Stable angina	527 (69.6)	680 (63.5)	**.007**
Congestive heart failure	152 (20.1)	197 (18.4)	.366
Peripheral arterial disease	85 (11.2)	174 (16.2)	**.003**
Hemoglobin A_1c,_ %	6.59 ± 0.05	6.57 ± 0.04	.578
Estimated glomerular filtration rate			
>60 L/min/1. 73 m^2^	547 (72.3)	778 (72.6)	.16
<60 mL/min/1. 73 m^2^ (average)	43.49 ± 0.91	46.03 ± 0.76	**.019**
Ejection fraction	0.5334 ± 0.42	0.5544 ± 0.36	**<.001**
Previous percutaneous coronary intervention/stent	135 (17.8)	222 (20.7)	.134
Percutaneous coronary intervention	125 (16.5)	205 (19.1)	.156
Stent	76 (10.0)	126 (11.8)	.257
Medications before coronary artery bypass grafting			
Aspirin	516 (68.2)	762 (71.1)	.177
P2Y12 receptor antagonist	100 (13.2)	309 (28.9)	**<.001**
Surgical variables			
On-pump surgery	511 (68.4)	836 (80.2)	**<.001**
Surgery duration, min	185.55 ± 56.89	209.87 ± 68.53	**<.001**
Medications after coronary artery bypass grafting			
β-blockers	667 (88.1)	928 (86.6)	.393
ACE-I/ARB	361 (47.7)	573 (53.5)	**.015**
Antiarrhythmic drugs	165 (21.8)	363 (33.9)	**<.001**
Calcium channel blocker	122 (16.1)	214 (20.0)	**.037**
Statin	660 (87.2)	970 (90.6)	**.027**
P2Y12 receptor antagonist	0	1071 (100)	**<.001**

Values are n (%) or mean ± SD. Bold *P* values are statistically significant.

ACE-I, angiotensin-converting enzyme inhibitor; AMT, aspirin monotherapy; ARB, angiotensin receptor blocker; DAPT, dual antiplatelet therapy.

DAPT was associated with significantly lower rates of all-cause mortality (odds ratio [OR], 0.43; 95% CI, 0.28-0.66; *P* < .001) and cardiovascular mortality (OR, 0.29; 95% CI, 0.16-0.56; *P* < .001) compared with AMT (Table 2). No statistically significant differences were observed in the incidence of post-CABG ACS (OR, 1.14; 95% CI, 0.71-1.85; *P* = .629), post-CABG CVA (OR, 0.80; 95% CI, 0.45-1.43; *P* = .457), or major adverse cardiac events (OR, 0.73; 95% CI, 0.52-1.01; *P* = .061).

**Table 2:** Primary and Secondary Outcomes

Variables	AMT	DAPT	Odds Ratio	95% CI	*P* Value
All-cause mortality	57 (7.5)	36 (3.4)	0.43	0.28-0.66	<.001
Cardiovascular mortality	34 (4.6)	15 (1.4)	0.30	0.16-0.56	<.001
Postcoronary artery bypass grafting					
Acute coronary syndrome	28 (3.7)	45 (4.2)	1.14	0.71-1.85	.629
Cerebrovascular accident	22 (2.9)	25 (2.3)	0.80	0.45-1.43	.457
Major adverse cardiac events	79 (10.6)	84 (7.9)	0.73	0.52-1.01	.061
Major bleeding	250 (33.0)	453 (42.3)	1.48	1.22-1.79	<.001
In-hospital blood transfusion	70 (9.5)	104 (10.1)	1.06	0.77-1.46	.710

Data are presented as n (%).

AMT, aspirin monotherapy; DAPT, dual antiplatelet therapy.

Overall survival rates were significantly higher in the DAPT group (97%) compared with the AMT group (93%; *P* < .001) (Figure, B). Furthermore, the cumulative survival rate remained higher in the DAPT group over each subsequent year of follow-up (Supplemental Table 1), with a similar follow-up time between the 2 groups (1004 ± 19.3 days vs 975 ± 16.1 days; *P* = .223) (Supplemental Table 1).

However, DAPT was associated with a significantly higher incidence of in-hospital postoperative major bleeding (OR, 1.48; 95% CI, 1.22-1.79; *P* < .001) compared with AMT, although there was no difference in transfusion requirements between the 2 groups (HR, 1.06; 95% CI, 0.77-1.46; *P* = .710) (Table 3). One patient in the DAPT group died of massive bleeding, whereas 2 patients in the AMT group and 1 in the DAPT group experienced pericardial hemorrhages causing tamponade.

**Table 3:** Multivariable Cox Regression Analysis for Predictors of Adverse Outcomes Post-CABG

Covariates	Hazard Ratio	95% CI	*P* Value
Age	1.06	1.54-3.15	<.001
Smoking status	1.58	1.12-2.23	.009
Chronic kidney disease	2.20	1.54-3.15	<.001
Ejection fraction	0.96	0.95-0.98	<.001
ACE-I/ARB	0.67	0.48-0.94	.021
Post-CABG P2Y12 receptor antagonist (DAPT group)	0.51	0.36-0.72	<.001

ACE-I, angiotensin-converting enzyme inhibitor; ARB, angiotensin receptor blocker; CABG, coronary artery bypass grafting; DAPT, dual antiplatelet therapy.

## Comment

Patients in the DAPT group were more likely to be smokers, were on DAPT before CABG, underwent more on-pump operations of longer duration, and had a history of peripheral arterial disease compared with those in the AMT group (Supplemental Results). Additionally, these patients were prescribed DAPT for variable duration (Supplemental Table 2). After adjustments for these factors, patients in the DAPT group exhibited significantly lower rates of all-cause mortality and cardiovascular mortality, with overall survival rates favoring DAPT at both short-and long-term follow-up.

Although there was no difference in major bleeding, except that DAPT was associated with a higher incidence of major postoperative bleeding, transfusion requirements did not differ between groups. Additionally, the Cox regression analysis identified important predictors of mortality, including older age, smoking, and chronic kidney disease, whereas higher ejection fraction, angiotensin converting enzyme–blocking drugs, and DAPT were protective factors (Supplemental Results). These results underscore the potential survival benefits of DAPT after elective CABG, which we observed in this study here in a large series of patients undergoing elective CABG.

Previous literature has demonstrated a clear survival benefit of DAPT in ACS patients after CABG, supported by large randomized controlled trials such as Clopidogrel in Unstable Angina to Prevent Recurrent Events (CURE), Trial to Assess Improvement in Therapeutic Outcomes by Optimizing Platelet Inhibition with Prasugrel-Thrombolysis in Myocardial Infarction (TRITON-TIMI 38), and Platelet Inhibition and Patient Outcomes (PLATO).^7^ However, evidence regarding DAPT’s role in elective CABG remains debatable. Some pooled data from randomized controlled trials investigating graft patency in stable ischemic heart disease patients exist but are limited by small sample sizes and have not adequately addressed mortality outcomes. Observational studies and meta-analyses, such as those by Agrawal and colleagues^8^ and Deo and colleagues^9^ have shown mixed results, with the latter emphasizing that DAPT benefits in CABG patients are largely driven by ACS populations, leaving a gap in understanding its impact on survival in elective CABG patients.

Our retrospective study provides new evidence in a large patient cohort suggesting a survival benefit from DAPT in patients undergoing elective CABG. This benefit likely stems from DAPT’s role in preventing early postoperative thrombosis, which is a major cause of acute graft occlusion.^10^ Given the similar pathophysiologic mechanisms between ACS and stable ischemic heart disease patients, DAPT’s ability to improve graft patency and reduce thrombosis may extend to elective CABG.

Although the superiority of DAPT over AMT in elective CABG is evident (Supplemental Figure), our study has certain limitations. Notably, the DAPT group had a higher number of on-pump operations, which may have contributed to the improved survival rates. Additionally, the study design prevented matching between the groups, which limits our ability to draw definitive conclusions based solely on these results.

Based on the results of this study and the observations, we believe that a large-scale randomized trial with an appropriate duration of DAPT after elective CABG would be beneficial and provide more definitive answers to this important question.
