# Supplementary material for: Dual Antiplatelet Therapy After Elective Coronary Artery Bypass Graft and Its Impact on Clinical Outcomes
Source: Ann Thorac Surg Short Rep. 2024 Dec 11;3(2):408–13. doi: 10.1016/j.atssr.2024.11.012 (PMC12167555; doi:10.1016/j.atssr.2024.11.012)
Supplement: Supplementary Material [file mmc1.docx]

**Tables and Figures:**

**Table 1. Event-Free Survival Rates**

| **Survival Rate** | **AMT** | **DAPT** | **p value** |
| --- | --- | --- | --- |
| 1-Year Event-Free Survival (%) | 94.3±0.9 | 98.9±0.3 | **<0.001** |
| 2-Year Event-Free Survival (%) | 93.1±1.0 | 97.9±0.5 | **<0.001** |
| 3-Year Event-Free Survival (%) | 92.5±1.0 | 96.4±0.7 | **<0.001** |
| 4-Year Event-Free Survival (%) | 91.9±1.1 | 95.5±0.8 | **<0.001** |
| 5-Year Event-Free Survival (%) | 90.5±1.5 | 94.4±1.1 | **<0.001** |

**Table 2.** Duration of Dual Antiplatelet Therapy

|  | **DAPT Duration (months)** | | | | | |
| --- | --- | --- | --- | --- | --- | --- |
|  | **1** | **1-3** | **3-6** | **6-12** | **≥ 12** | **Unknown** |
| **Count (n)** | 18 | 416 | 76 | 99 | 452 | 10 |
| **Percentage (%)** | 1.7% | 38.8% | 7.1% | 9.2% | 42.2% | 0.9% |

DAPT= Dual antiplatelet therapy, n= number

**
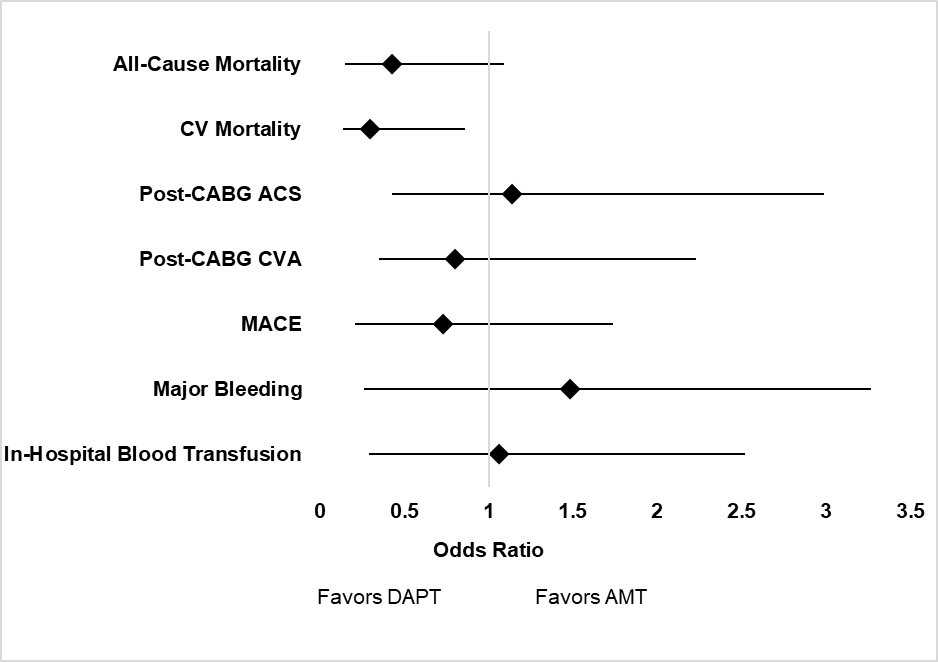
**

**Figure 1: Forest Plot.**

**Results:**

**Patient Characteristics:**

A total of 3,900 patients who underwent CABG surgery between 2012 and 2015 at the Oklahoma Heart Hospital (Oklahoma City, OK, USA) were reviewed. After applying the specified exclusion criteria, 1,828 patients who had undergone surgery for SIHD were identified and included in this study. Among them, more patients were prescribed DAPT (n=1,071; 58.6%) than AMT (n=757; 41.4%). The baseline characteristics of the patients in both treatment groups are summarized in Table 1. Patients receiving DAPT after CABG tended to be younger and had a lower incidence of chronic kidney disease and peripheral arterial disease than those receiving DAPT after AMT. Additionally, DAPT patients were more likely to be smokers and had a history of receiving DAPT before CABG. There were no significant differences between the groups in the average number of grafts, number of arterial grafts, or other surgical variables. Patients on DAPT were more likely to have undergone on-pump surgery and had longer surgical times than those on AMT. Patients in the DAPT group were more frequently administered antiarrhythmic drugs, calcium channel blockers, and ACE inhibitors postoperatively. Patients who died within 48 hours of surgery (n=11) were excluded from subsequent analyses. Approximately 42% of patients received DAPT for more than 12 months. A breakdown of the DAPT cohort according to treatment duration provided in Table 4.

**Regression analysis:**

The hazard ratio (HR) of the covariates, including pre-CABG variables (i.e., age, smoking status, chronic kidney disease (CKD), stable angina status), peripheral arterial disease (PAD), ejection fraction, P2Y12 receptor antagonist), surgical variables (i.e., on-pump surgery, surgery duration), and post-CABG medications (i.e., angiotensin-converting enzyme inhibitor or angiotensin receptor blocker (ACE-I/ARB), antiarrhythmic drugs (AAD), calcium channel blocker (CCB), statin, P2Y12 receptor antagonist), were estimated using the Cox regression model. The post-CABG P2Y12 receptor antagonist (i.e., AMT vs. DAPT groups), age, smoking status, CKD, EF and AAD were included in the equation. The hazard ratio for the post-CABG P2Y12 receptor antagonist group was estimated at 0.51 (95% CI 0.36-0.72; p<0.001); the DAPT group had half the risk of all-cause mortality as the AMT group. Age (HR=1.06, 95% CI 1.04-1.08; p<0.001), smoking status (HR=1.58, 95% CI 1.12-2.23; p=0.009), and CKD status (HR=2.20, 95% CI 1.54-3.15; p<0.001) were associated with a greater risk of all-cause mortality, while higher EF (HR=0.96, 95% CI 0.95-0.98; p<0.001) and ACE/ARB use after CABG (HR=0.67, 95% CI 0.48-0.94; p=0.021) were associated with a lower risk of all-cause mortality. The estimated hazard ratio of on-pump surgery was analyzed using the Cox regression model, which revealed that on-pump surgery was not a significant factor contributing to the survival rate (p=0.792).
